# Prevalence and risk factors of human papilloma virus infection among women living with HIV, Egypt, a cross sectional study

**DOI:** 10.1186/s12889-024-19240-z

**Published:** 2024-07-09

**Authors:** Mona Ashry, Shaimaa Shawky, Zeinab Mounir, Fawzy Fathy, Heba Elsayed, Walid Kamal, Mohamed Hassany

**Affiliations:** 1 Faculty of Medicine, (Public Health Department), Alexandria University, Alexandria, Egypt; 2 Arab Academy for Science, Technology & Maritime Transport, College of Medicine, Alamein City, Egypt; 3 Central Public Health Laboratory, Ministry of Health & Population, Cairo, Egypt; 4 National AIDS Program, Cairo, Egypt; 5 Mother and Child Health Directorate, Ministry of Health & Population, Cairo, Egypt; 6 UNAIDS Country Director, Cairo, Egypt; 7 Minister Assistant for Public Health Initiatives and Projects, Ministry of Health & Population, Cairo, Egypt; 8 National Hepatology & Tropical Medicine Research Institute (NHTMRI), Cairo, Egypt

**Keywords:** Women living with HIV, Human papilloma virus, Egypt

## Abstract

**Background:**

HPV is considered the most common sexually transmitted infection. It is responsible of 70% of cervical cancers worldwide. HIV infection is associated with increased rates of HPV infection. Women Living With HIV (WLWH) are 6 times at greater risk of developing cervical cancer. The current study aimed to estimate prevalence and identify genotypes of HPV infection among WLWH in Egypt compared to women with negative HIV status and determine associated risk factors.

**Methods:**

The study conducted among 251 WLWH and 268 women with negative HIV status enrolled from gynecological clinics in primary health care centers from nine Egyptian governorates. Data was collected from participants using a structured interview questionnaire and cervical samples were collected for HPV DNA detection and genotyping.

**Results:**

The overall prevalence of HPV infection was 13.5%, 3.4% among women with HIV negative status and 24.4% among WLWH. HR-HPVs other than genotype 16 and 18 were isolated from 71% of infected women. Woman’s age, age at first marriage, number of lifetime marriages and drug addiction are significant predictors for HPV infection (odds 0.96, 0.91, 2.06, 2.01 respectively).

**Conclusion:**

HPV infection is more prevalent among WLWH. Infection with HR-HPV other than genotype 16 and 18 was the most prevalent among infected women in both groups. Young age, early life sexual activity, having more than one sexual partner during the life time, and drug addiction are independent predictors for HPV infection. Having a husband who has had other sexual partners is significantly associated with infection.

## Background

Cervical cancer is considered the 4th most common cancer in women. The HPV (Human Papilloma Virus) information center in 2023 estimated the worldwide incidence rate of cervical cancer among women in the reproductive age to be 13.1 per 100,000 women [1]. In 2020, the estimated number of women diagnosed with cervical cancer globally was 604,000 women with estimated 342,000 deaths from the disease [2]. In Egypt, the World Health Organization (WHO) estimated the crude incidence rate of cervical cancer in women to be 2.6/ 100,000 women in 2020 with a total 620 fatalities from the disease in 2019 [3].

Early in 20th century a relationship was suspected between cervical cancer and sexual behavior which then confirmed in 1960s. In 1980s, HPV was detected in cervical cancer cells. This finding was the starting point for further research which confirmed the presence of a consistent association between HPV and cervical cancer where nearly all cases of cervical cancer are found to be due to chronic HPV infection [4–6].

HPV is considered the most common sexually transmitted viral infection. There are more than 170 HPV genotypes among them 15 types are recognized as high risk (HR HPV) or cancer-causing genital HPVs. HPV type 16 and 18 are considered the most prevalent types detected in HPV associated cancers. They were found to be responsible of about 70% of cervical cancers worldwide [7, 8].

HIV is associated with increased rates of HPV infection. In addition of being both sexually transmitted infection, HIV positive status facilitates HPV persistence after acquisition of infection and modulates the expression of HPV E6 and E7 genes which are responsible of oncogenic transformation. It was found that women living with HIV(WLWH) are 6 times at greater risk of developing cervical cancer [9, 10].

The Middle East and North Africa region (MENA) involves many countries and territories which are markedly varied regarding their demographic trends, dynamics and gross national income. Urban and young population is dominating in the region with more than half of the population is under the age of 25. Islam is the dominating religion in the region. Egypt is considered one of the region’s middle-income countries [11, 12]. Although the Middle East and North Africa is the region of the lowest HIV burden in the world with 190,000 People living with HIV (PLWH) in 2022, the number of new HIV infections increased by 33% from year 2010 to 2021 and reach 61% increase by year 2023 making the HIV epidemic in the region on the rise. This increase is driven primarily by transmission among population with high-risk behaviors and their sexual partners who estimated to have accounted for 85% of those new infections. These numbers represent only people who presented themselves for testing as the region is still facing the problem of underdiagnosis and inefficient testing. In year 2022, it was found that only 67% of PLWH knew their HIV status. In Egypt, it is estimated that there was 34,000 PLWH in 2022, among them there were 5700 WLWH [13]. HPV Vaccine is not yet introduced in the Egyptian national immunization program however it is introduced in the private sector since 2009 to those who desire and can afford the cost. Moreover, till date, there is no Egyptian national cervical cancer screening program [3]. Focusing on the prevention and treatment of HIV and cervical cancer, integration of services will maximize the benefits [14].

In 2020, WHO launched the Global strategy to accelerate the elimination of cervical cancer as a public health problem. The strategy has three main global targets for 2030 including “90% of girls are fully vaccinated with HPV vaccine by age 15 years, 70% of women are screened with a high-performance test by 35 years of age and again by 45 years of age, 90% of women identified with cervical disease receive treatment”. The currently available HPV vaccines include bivalent vaccines (HPV 16 & 18), quadrivalent vaccine where genotypes 6 and 11 added and the 9-valent vaccine including, in addition, genotypes 31, 33, 45, 52 and 58. Based on the Global strategy targets, WHO updated its guidelines with inclusion of 16 recommendations and good practice statements for WLWH. The key features of these recommendations include HPV DNA detection for primary screening starting at the age of 25 years with regular screening every 3 to 5 years; partial genotyping, colposcopy or cytology should be carried out to triage women who tested positive” triage a second test for positive cases”; and treatment of detected precancerous lesions and histologically confirmed adenocarcinoma in situ through large-loop excision of the transformation zone or cold knife conization [14].

On preparation for launching of the Egyptian presidential initiative for early detection of cancers including cancer cervix, this study was conducted aiming to provide an estimate for HPV prevalence - as the main cause for occurrence of cancer cervix- among women living with HIV compared to women with negative HIV status in Egypt, identify the types of HPVs isolated from infected women and determine risk factors associated with infection.

## Methods

A cross sectional study was conducted among a convenient sample of WLWH and women with negative HIV status who were recruited from nine Egyptian governorates representing the different geographical regions, cultures, behaviours, norms and traditions in Egypt namely Cairo, Giza, Alexandria, Al-Qalyubia, Dakahlia, Gharbia, Sharkia, Fayoum and AL-Minia.

By using Epi Info software for sample size calculation and based on 48% estimated prevalence of HPV infection among WLWH and 28% among HIV negative women [15], with equal allocation of women in both groups, the minimum sample size required in each group was 65 women.

Based on the calculated sample size, a percentage for the number of women recruited from each governorate was allocated so as to respect weighing as per the number of Women living with HIV registered in each governorate. Data was collected at the gynaecological clinics in primary health care centres at these nine governorates. Target population included women aged 18 to 50 years old attending those clinics and WLWH who were referred to those clinics from the HIV care centres during the four months period of the field work (from March to June 2023) and agreed to provide a sample for HPV testing. Exclusion criteria included having abundant menstrual bleeding or vaginal discharge not allowing appropriate screening to be performed, history of hysterectomy, known diagnosis of immunosuppression or patient on immunosuppressive medications, pregnant women, having received one or more doses of HPV vaccine, and having any gynaecological cancer.

A capacity building program was designed and implemented to train all physicians and nurses who are responsible for providing care at the study settings on the designed data collection tool and methodology of cervical sample collection for HPV DNA detection. Women who attended the clinics during the period of the field work; fulfil the inclusion and exclusion criteria and voluntarily accepted participation in the study; were exposed to pregnancy testing to confirm being non pregnant and HIV testing for the control group to confirm being negative before enrolment in the study.

All participants were interviewed by the clinic physician using the designed structured interview questionnaire to collect data regarding their sociodemographic characteristics, reproductive data and risk factors for sexually transmitted infections. Women were instructed at least 48 h before taking the cervical smear sample to void, cleaning the genital area by using vaginal douches and using the condom if there was any sexual practice.

Cervical smear samples were collected using a brush inserted into the cervix, pushed gently and rotated five times in a clockwise direction, rinsed as quickly as possible ten times into the solution vial then swirled vigorously and discarded. The cap was tightened so that the torque line on the cap passes the torque line on the vial and stored at room temperature. HPV DNA detection was carried out in the central laboratories affiliated to the Egyptian Ministry of Health. The type of the HPV test used was HPV PCR pap smear test (Qualitative nucleic acid test for use on the cobas^®^ฏ 6800/8800 Systems For in vitro diagnostic use). Intended use Cobas^®^ฏ HPV for use on the Cobas^®^ฏ 6800/8800 Systems (cobas^®^ฏ HPV) is a qualitative in-vitro test for the detection of Human Papillomavirus in clinician-collected cervical specimens using an endocervical brush/spatula or broom and placed in the ThinPrep^®^ฏ Pap Test™ฏ PreservCyt^®^ฏ Solution. This test detects the high-risk HPV types 16, 18, 31, 33, 35, 39, 45, 51, 52, 56, 58, 59, 66, and 68. IT detects HPV16 on separate channel, HPV18 on separate channel, and other high-risk HPV mentioned above all on one channel. Data was coded then entered and analysed using IBM SPSS statistics version 20. Categorical variables were presented as number and percentage. For quantitative variables, minimum, maximum, mean and standard deviation were used. Comparisons of studied variables between both study groups (WLWH and control group) were carried out, then according to results of HPV PCR pap smear testing, the studied women were further divided into HPV positive and negative groups. Comparisons between groups were carried out using Chi Square test for categorical variables. If one or more cells in the table has an expected count less than 5Fisher’s Exact test was used for 2 × 2 tables and Monte Carlo test for other contingency tables. For normally distributed quantitative variables, student t test was used while Mann-Whitney U test was used for abnormally distributed variables. Multiple variate logistic regression analysis was used to assess the independent predictors of HPV infection. Chi square test was used to asses significance of the model and odds ratio was used as a measure of risk. All results were interpreted at 5% level of significance.

## Results

The study included 519 women, 251 women had laboratory confirmed HIV infection and 268 women with negative HIV status. The study sample were collected from nine governorates representing the main characteristics of different Egyptian governorates as presented in Table 1. The sociodemographic characteristics of studied women are presented in Table 2.

**Table 1:** Distribution of studied women based on their HIV status and residence

Governorate	Cases WLWH *n* = 251 *n* (%)	Control group *n* = 268 *n* (%)
Cairo	66 (26.3)	69 (25.7)
Giza	40 (15.9)	40 (14.9)
Alexandria	40 (15.9)	40 (14.9)
Al Qalyubia	25 (10)	25 (9.3)
Dakahlia	20 (8)	26 (9.7)
Gharbia	20 (8)	21 (7.8)
Sharkia	20 (8)	20 (7.5)
Fayoum	10 (4)	17 (6.3)
Minia	10 (4)	10 (3.7)

**Table 2:** Distribution of studied women based on their HIV status and sociodemographic characteristics

Characteristics	Cases (WLWH) *n* = 251	Control Group *n* = 268	Test of significance
Age (years) Min-Max Mean ± SD	19–50 35.53 ± 7.1	18–50 34.67 ± 7.73	Student t test t = 1.31 *p* = 0.19
Age at first marriage (years) < 18 years old ≥ 18 years old	59 (23.5) 192 (76.5)	28 (10.4) 240 (89.6)	Chi Square X^2^ = 8.4 *P* = 0.001* Odd’s = 2.63 (1.62–4.29)
Years of marriage Min-Max Mean ± SD	1–34 14.95 ± 7.09	1–33 13.07 ± 8.06	Student t test t = 2.83 *p* = 0.005* Mean difference = 1.88 (0.58–3.19)
Number of marriages Min-Max Mean ± SD	1–4 1.28 ± 0.58	1–3 1.06 ± 0.27	Student t test t = 5.49 p ˂0.001* Mean difference = 0.22 (0.14–0.3)
Residence - Urban - Rural - Slummy area	175 (69.7) 71 (28.3) 5(2%)	216 (80.6) 48 (17.9) 4(1.5)	Monte Carlo *P* = 0.015* (0.012 − 0.018)
Education - Illiterate - Primary school - Preparatory school - Secondary school - Institute graduate - University graduate	38 (15.1) 38 (15.1) 37 (14.7) 94 (37.5) 11 (4.4) 33 (13.1)	21 (7.8) 17 (6.3) 47 (17.5) 123 (45.9) 15 (5.6) 45 (16.8)	Chi Square X^2^ = 19.9 *P* = 0.001* (0.000–0.002)
Occupation - Housewife - Paid work - Others	177 (70.5) 74 (29.5) 0	215 (80.3) 51 (19) 2 (0.7)	Monte Carlo *p* = 0.006* (0.004–0.007)

*Statistically significant at *p* ≤ 0.005

HPV infection screening test showed valid laboratory results for 246 WLWH (5 cases had invalid lab results) and for 264 women in the control group (4 women had invalid lab results). The overall prevalence of high-risk HPV infection among studied women was 13.5% (*n* = 69). The estimated prevalence of high-risk HPV infection among studied WLWH was 24.4% (*n* = 60) compared to only 3.4% (*n* = 9) among the control group and this difference is statistically significant, (Chi square test X^2^ = 47.961, P˂0.001). Table 3 shows the genotypes of HPVs isolated from positive samples.

**Table 3:** Distribution of HPV infected women based on type of isolated HPVs

Genotype of HPV isolated	Cases (WLWH) *n* = 60	Control group *n* = 9
HPV 16	5 (8.3)	2 (22.2)
HPV 18	0	1 (11.1)
Other HR-HPV	44 (73.4)	5 (55.6)
Mixed infection	11 (18.3)	1 (11.1)

On studying the characteristics of women with and without HPV infection, it was revealed that the mean age of HPV infected women (33.43 ± 6.38) was less than the age of women with negative HPV test result (35.35 ± 7.59) and the difference is statistically significant (*p* = 0.04). Moreover, women with HPV infection had significantly lower age of marriage and greater number of marriages compared to women with negative HPV test results (*p* = 0.001 & ˂0.001 respectively). There is no statistically significant difference between both studied groups regarding residence, education, years of marriage, history of abortion and use of Intrauterine Device (IUD). (Table 4)

**Table 4:** Distribution of studied women based on their sociodemographic & reproductive characteristics and laboratory results of HPV screening

Women Characteristics	HPV infection screening		Test of significance
	Positive *n* = 69	Negative *n* = 441	
Age (years) Min-Max Mean ± SD	19–46 33.43 ± 6.38	18–50 35.35 ± 7.59	Student t test t = 1.99 *p* = 0.04* Mean difference = 1.91 (0.02–3.81)
Residence - Urban - Rural - Slummy area	53 (76.8) 15 (21.7) 1 (1.5)	329 (74.6) 104 (23.6) 8 (1.8)	Monte Carlo *P* = 0.9
Education - Illiterate - Primary school - Preparatory school - Secondary school - Institute graduate - University graduate	10 (14.5) 9 (13.1) 13 (18.8) 24 (34.8) 2 (2.9) 11 (15.9)	49 (11.1) 46 (10.4) 68 (15.4) 191 (43.3) 22 (5) 65 (14.8)	Monte Carlo *P* = 0.69
Age at first marriage Min-Max Mean ± SD	13–31 19.6 ± 3.6	11–38 21.37 ± 4.2	Student t test t = 3.31 *p* = 0.001* Mean difference = 1.76 (0.72–2.81)
Years of marriage Min-Max Mean ± SD	1–28 13.83 ± 6.56	1–34 13.98 ± 7.58	Student t test t = 0.152 *p* = 0.88
Number of marriages Min-Max Mean ± SD	1–4 1.73 ± 0.69	1–4 1.13 ± 0.39	Mann-Whitney U test Z=-3.956 p ˂0.001* Mean rank for HPV positive group = 294.68 Mean rank for HPV negative group = 249.37
History of abortion - Yes - No	12 (17.4) 57 (82.6)	62 (14.1) 379 (85.9)	Chi square X^2^ = 0.534 *P* = 0.47
History of IUD use - Yes - No	25 (36.2) 44 (63.8)	199 (45.1) 242 (54.9)	Chi square X^2^ = 1.916 *P* = 0.16

*Statistically significant at *p* ≤ 0.05

Studying the risky behaviors that are associated with sexually transmitted diseases, it was found that husband’s sexual relation with other women and women or husband drug addiction have statistically significant association with HPV infection (p˂0.001, 0.002 respectively). (Table 5)

**Table 5:** Distribution of studied women based on their laboratory results of HPV screening and risk factors for sexually transmitted infections

Studied variable	HPV infection screening		
	Positive *n* = 69	Negative *n* = 441	Test of significance
Husband has other sexual relations - Yes - No - Do not know	17 (24.6) 30 (43.5) 22 (31.9)	43 (9.8) 312 (70.7) 86 (19.5)	Chi Square X^2^ = 22.18 P˂0.001* (0.000–0.001)
Having sexual relations before marriage - Yes - No	*n* = 64 2 (3.1) 62 (96.9)	*n* = 426 3 (0.7) 423 (99.3)	Fisher’s Exact *p* = 0.13
Wife or husband drug addiction - Yes - No	28 (40.6) 41 (59.4)	102 (23.1) 339 (76.9)	Chi Square X^2^ = 9.273 *P* = 0.002* Odd’s = 2.24 (1.32–3.8)

*Statistically significant at *p* ≤ 0.05

On multiple logistic regression analysis, women’s age, age at first marriage, number of marriages and women and/or husband drug addiction were significant predictors of HPV infection. Table 6 shows that every one-year increase in woman’s age, women are 4% less likely to acquire HPV infection. Every one-year increase in the age at first marriage, women are 9% less likely to acquire HPV infection. Every one unit increase in the number of marriages (reflecting lifetime sexual partners and sexual activity), the odds of HPV positive infection status will increase by 2.069 (the risk of acquiring HPV infection increases by 207%). Being a drug addict or having a husband who is drug addict, will increase the odds of HPV positive infection status by 2.01 (the risk of acquiring infection increases by 201%).

**Table 6:** Multiple logistic regression analysis of independent predictors of HPV infection

Independent predictors	B	Significance	Odds ratio (95% Confidence Interval)
Age	-0.038	0.04	0.96 (0.92–0.99)
Age at first marriage	-0.86	0.03	0.91 (0.84–0.99)
Number of marriages	0.727	0.003	2.069 (1.29–3.33)
Drug addiction (women and/or husband)	0.7	0.01	2.01 (1.15–3.53)

Model Chi square X^2^ = 31.783, *P* = 0.000; Constant: -1.855, *p* = 0.000

## Discussion

Sexually active women and men are found to be infected at least once with HPV during their life time making HPV infection the most common sexually transmitted infection worldwide. Although HPV infection is frequently not associated with any pathologies, it is considered the primary cause of female cancers. It is isolated from up to 90% of cervical cancers making it a public health priority for surveillance and monitoring [16, 17].

Regarding the prevalence of HPV infections, Asia and Africa have the highest prevalence among the world continents. Within continent, prevalence of infections is higher in developing compared to developed regions [18]. The current study which is carried out among women from nine governorates representing the different regions of Egypt regarding geographical; social and cultural characteristics; the overall estimated prevalence of infection was 13.5% regardless of the HIV status of studied women. Previous studies carried out in Egypt from year 2006 to 2018 showed a varied prevalence ranging from 10.3% up to 23.1% [19–21]. The differences in estimated prevalence between these studies could be attributed to the characteristics of studied women where studies with higher prevalences [19, 21] were conducted among women enrolled from Cairo which is the capital city while the lowest was from a multicenter study [20] conducted among women enrolled from tertiary care hospitals with wide catchment areas. According to biological and behavioral surveillance data in Egypt, risky behaviors are concentrated in Cairo and Alexandria -which are the biggest Egyptian cities- and reflected on the concentrated number of HIV cases among injecting drug users and men who have sex with men in these cities [22].

With focus on the HPV co-infection with HIV, the present study showed a significantly higher prevalence of infection among HIV positive (24.4%) compared to HIV negative women (3.4%). This is similar to results of studies conducted in many other countries worldwide and attributed to the reduced clearance rate and increased persistence of HPV infection in HIV positive women [9, 23–25].

Infection with HR-HPVs other than HPV 16 and 18 represents the majority of isolates among both studied groups (71%) followed by mixed infections (17.3%). Solitary infection with HPV16 or 18 represents only around one tenth of infections (11.6%) and was more frequent among women with HIV negative status. This is similar to results reported from other studies conducted in other regions [23, 26]. This finding was expected as HPVs share the same mode of transmission. Also, generated HIV immune responses during seroconversion and associated immunosuppression favor low pathogen clearance, reactivation of HPV latent stage infections and persistence of multiple types of HPVs. Moreover, non-HPV-16 genotypes; which are less efficient at evading the immune system; use the advantage given by HIV associated state of immunosuppression resulting in decrease clearance rate and increased persistence of these infections [27]. Also, it should be taken into consideration that this difference may be attributed to the gathering of HR-HPV other than HPV 16 and 18 in one category based on the used laboratory technique. So, further working on estimating the actual prevalence of individual HR-HPV to identify the most circulating types is recommended with inclusion of more HR-HPV genotypes in the produced HPV vaccines according to these results.

As age is an important determinant in the risk of acquiring HPV infection, the present study revealed that the mean age of HPV positive women is significantly lower than HPV negative women. This finding is on agreement with the fact that HPV infection is most common among sexually active young women with marked decline in prevalence after the age of 30 years [28].

Sexual activity is an important risk for acquiring HPV infection. Sexual activity at an early age, had multiple sexual partners at any time in life and having a partner who has had multiple sexual partners increase the risk of HPV infection [28]. These explain the significant association found between HPV infection and early age of marriage, multiple marriages which reflects the number of lifetime sexual partners and activity and having a husband who has had other sexual partners.

Mechanical disruption of the stratified squamous epithelium of the cervix caused by abortions and insertion of IUDs was found to facilitate HPV virus access to basal cells [29]. But the results of the current study showed insignificant association between history of abortion or use of IUDs and HPV infection and this could be attributed to the small sample size studied in these categories.

The significant association between drug addiction and HPV infection revealed in the current study and other studies is attributed to the high-risk sexual behavior encountered among drug addicts which makes it a common factor associated with both HIV and HPV infection.

Multiple logistic regression analysis revealed that variables related to sexual behavior are the main independent predictors of HPV infection including young age, early age of sexual activity reflected by the early age of marriage, having more than one lifetime sexual partner reflected by multiple marriages and lastly drug addiction which commonly associated with high-risk sexual behaviors.

## Conclusion

HPV infection is significantly more prevalent among women living with HIV. Infection with HR-HPVs other than genotype 16 and 18 is the most prevalent among Egyptian women both WLWH and women with HIV negative status. Early marriage, having more than one lifetime sexual partner reflecting high sexual activity and increased exposure risk, having a husband who has had other sexual partners are associated with increased women’s risk of acquiring HPV infection. Drug addiction; due to the associated high risk sexual behavior; is found to be a significant risk factor for HPV infection.

## Recommendation

Future research work is recommended to estimate the actual prevalence of individual HR-HPVs and consider results in including more HR-HPV genotypes in the HPV vaccine. Although the low estimated prevalence of HPV infection among Egyptian women with HIV negative status, discussions on the introduction of HPV vaccination in the national immunization program for young girls and boys is recommended in order to prevent infection associated cancers and mortality which have a dramatic sequalae on the social life. Integration of HPV screening and early detection of cervical cancers with HIV services will target the population of the highest risk in a cost-effective manner with maximization of benefits. Drug addiction prevention and control programs should be a public health priority as it will have an indirect effect in prevention of sexually transmitted infections including HIV and HPV.

## Data Availability

The data that support the findings of this study are available from [Egyptian Ministry of Health & Population] but restrictions apply to the availability of these data, which were used under license for the current study, and so are not publicly available. Data are however available from the authors upon reasonable request and with permission of [Egyptian Ministry of Health & Population].

## Abbreviations

HIV: Human Immunodeficiency Virus
HPV: Human Papilloma Virus
HR-HPV: High risk Human Papilloma virus
IUDs: Intrauterine devices
MENA: Middle East and North Africa
PCR: Polymerase Chain Reaction
WHO: World Health Organization
WLWH: Women living with HIV
